# Developing Machine Learning Algorithms to Predict Pulmonary Complications After Emergency Gastrointestinal Surgery

**DOI:** 10.3389/fmed.2021.655686

**Published:** 2021-08-02

**Authors:** Qiong Xue, Duan Wen, Mu-Huo Ji, Jianhua Tong, Jian-Jun Yang, Cheng-Mao Zhou

**Affiliations:** ^1^Department of Anesthesiology, Pain and Perioperative Medicine, The First Affiliated Hospital of Zhengzhou University, Zhengzhou, China; ^2^Department of Anesthesiology, The Second Affiliated Hospital, Nanjing Medical University, Nanjing, China

**Keywords:** machine learning, pulmonary complications, diffuse peritonitis, predict, AUC

## Abstract

**Objective:** Investigate whether machine learning can predict pulmonary complications (PPCs) after emergency gastrointestinal surgery in patients with acute diffuse peritonitis.

**Methods:** This is a secondary data analysis study. We use five machine learning algorithms (Logistic regression, DecisionTree, GradientBoosting, Xgbc, and gbm) to predict postoperative pulmonary complications.

**Results:** Nine hundred and twenty-six cases were included in this study; 187 cases (20.19%) had PPCs. The five most important variables for the postoperative weight were preoperative albumin, cholesterol on the 3rd day after surgery, albumin on the day of surgery, platelet count on the 1st day after surgery and cholesterol count on the 1st day after surgery for pulmonary complications. In the test group: the logistic regression model shows AUC = 0.808, accuracy = 0.824 and precision = 0.621; Decision tree shows AUC = 0.702, accuracy = 0.795 and precision = 0.486; The GradientBoosting model shows AUC = 0.788, accuracy = 0.827 and precision = 1.000; The Xgbc model shows AUC = 0.784, accuracy = 0.806 and precision = 0.583. The Gbm model shows AUC = 0.814, accuracy = 0.806 and precision = 0.750.

**Conclusion:** Machine learning algorithms can predict patients' PPCs with acute diffuse peritonitis. Moreover, the results of the importance matrix for the Gbdt algorithm model show that albumin, cholesterol, age, and platelets are the main variables that account for the highest pulmonary complication weights.

## Introduction

Complex intra-abdominal infections may result in localized or diffuse peritonitis (1). Thus, early prognostic assessment and testing for diffuse peritonitis is essential for assessing disease severity and optimizing treatment (2). Studies have shown that the mortality rate for patients with diffuse peritonitis is 9% (3).

Postoperative pulmonary complications (PPCs) are a major cause of morbidity after upper abdominal surgery, as they lengthen hospital stays and increase medical costs (4). PPC refers to postoperative pulmonary abnormalities with clinical manifestations and negative effects on disease progression with an incidence of 10–30%. Examples of PPCs include atelectasis, pulmonary infections, pleural effusion, and pulmonary thromboembolism (5–7). In clinical application, vital capacity is used for risk assessment of pulmonary complications, but critical state of lung function cannot predict complications. Moreover, there are a variety of interventions which prevent pulmonary complications, including a pre-emptive strategy to optimize respiratory physiology, and interventions during and after surgery. However, due to multiple factors pertaining to strategy, it is impossible to confirm which part of the intervention is the most important. Therefore, there is an urgent need for more effective measures and new technologies for predicting and preventing postoperative pulmonary complications.

Recent years have seen a growing body of research on machine learning and perioperative medicine (8–11). Machine Learning (ML) methods can predict clinical outcomes better than traditional statistical methods (12). For example, Fei et al. (13) used clinical data on severe acute pancreatitis (SAP) to construct Artificial Neural Network (ANN) and logistic regression models. Nijbroek et al. have argued that machine learning can support the development of more powerful PPC prediction models (14). However, studies have shown that machine learning has no performance advantage over logistic regression for clinical prediction models (15).

The present study explores the use of machine learning to improve the prediction of postoperative pulmonary complications in patients with diffuse peritonitis.

## Materials and Methods

### Patients

#### Ethics Committee Approval and Consent to Participate

This is a secondary data analysis using database data. Data are available from the BioStudies (public) database (https://www.ebi.ac.uk/biostudies/studies?query=S-EPMC6034864). In accordance with local laws and institutional requirements, ethical review and approval was not required for this study on human participants. In accordance with national laws and institutional requirements, written informed consent was not required from patients to participate in this study.

The data included medical records from critically ill patients who had received emergency gastrointestinal surgery for diffuse peritonitis.

### Perioperative Variables

The following variables are included in the analysis: body mass index, sex, age, ASA score, lesion location, diagnosis, perioperative shock, preoperative laboratory findings, postoperative complications (3), and type of surgery.

### Machine Learning Algorithms

The aim of classification by logistic regression is to establish a regression formula to classify boundary lines based on existing data. Logistic regression is a linear fit of a response variable to a logarithmic probability ratio. The coefficients are obtained by maximum likelihood estimation. The intuitive meaning of the maximum likelihood is that a pair of estimates for B0 and B1 is needed to predict the probability of the observations they produce, as close as possible to the actual observation of Y (the likelihood). A linear regression model is expressed as an equation that calculates a particular weight (i.e., coefficient b) for the input variable, and then describes a straight line that best fits the relationship between the input variable (x) and the output variable (y). For example: y = B0 + B1 ^*^ x.

Decision tree learning is a decision model that incorporates data attributes into a tree structure. Decision trees are often constructed based on a given dataset (16). Decision tree algorithm is a method of approximating discrete function values. It is a typical classification method, which first processes the data, generates readable rules and decision trees using inductive algorithms, and then uses the decisions to analyze the new data. In short, decision tree is a process for classifying data based on a series of rules.

The Gradient Boosting Decision Tree (GBDT) method (17) is used for data bulletins, to create M models (such as classification). This model is simple, and it is referred to as a weak learner. For each classification, the weight of the data incorrectly divided the previous time is increased one point before classification. In this way, the final classifier can produce good results, for both test data and training data.

Lightgbm (gbm) is another implementation of GBDT (18). Based on GBDT, it adopts two new strategies.

Our analysis was conducted with R version 3.1.3 (http://www.R-project) and Python version 3.6 (Python Software Foundation). We used five machine learning algorithms (Logistic regression, DecisionTree, GradientBoosting, Xgbc, and gbm) (19, 20) to predict postoperative pulmonary complications. We randomly divided all samples into training and test groups at a ratio of 7:3 using 5-fold cross-validation. We performed the 5-fold cross-validation in the training group, and then obtained its optimal model and parameters, and applied them to the test group. Five-fold cross-validation is a data splitting strategy for cross-validation, that is, the data set is split into A data set and B data set. The principle is: First, the whole training data set is divided into 5-folds, where 4-folds are used as the A data set to train the model, and the remaining 1-fold is used as the B data set to score the model, and the above process is repeated five times. In the weighted correlation analysis, we ranked the variables from highest to lowest weighted scores accounting for pulmonary complications. We did this using Pearson correlation analysis. The variables' missing values are supplemented by multiple imputation. The values were normalized and scaled 0–1. ROC is an abbreviation for “receiver operating characteristic.” The ROC curve's area is the AUC (Area Under the Curve). AUC (area under the ROC curve), i.e., the area under the ROC curve, the larger the better, indicating that the model had higher prediction value: (1) AUC≈1.0: the most ideal test index; (2) AUC is within 0.7–0.9: the model has high accuracy; (3) AUC ≤ 0.5: the model has no predictive value. We can only provide code that runs out of the results portion of the algorithm because of the patent application issues involved. See Appendix 1 for specific codes.

## Results

Nine hundred and twenty-six cases were included in this study; 187 cases (20.19%) had postoperative pulmonary complications. The average age of the patients with postoperative pulmonary complications was 65.6 (± 14.5) years old. One hundred and twenty-six (67.4%) were males with postoperative pulmonary complications, and 61 (32.6%) were females with postoperative pulmonary complications (see Supplementary Table 1).

**Figure 1:**
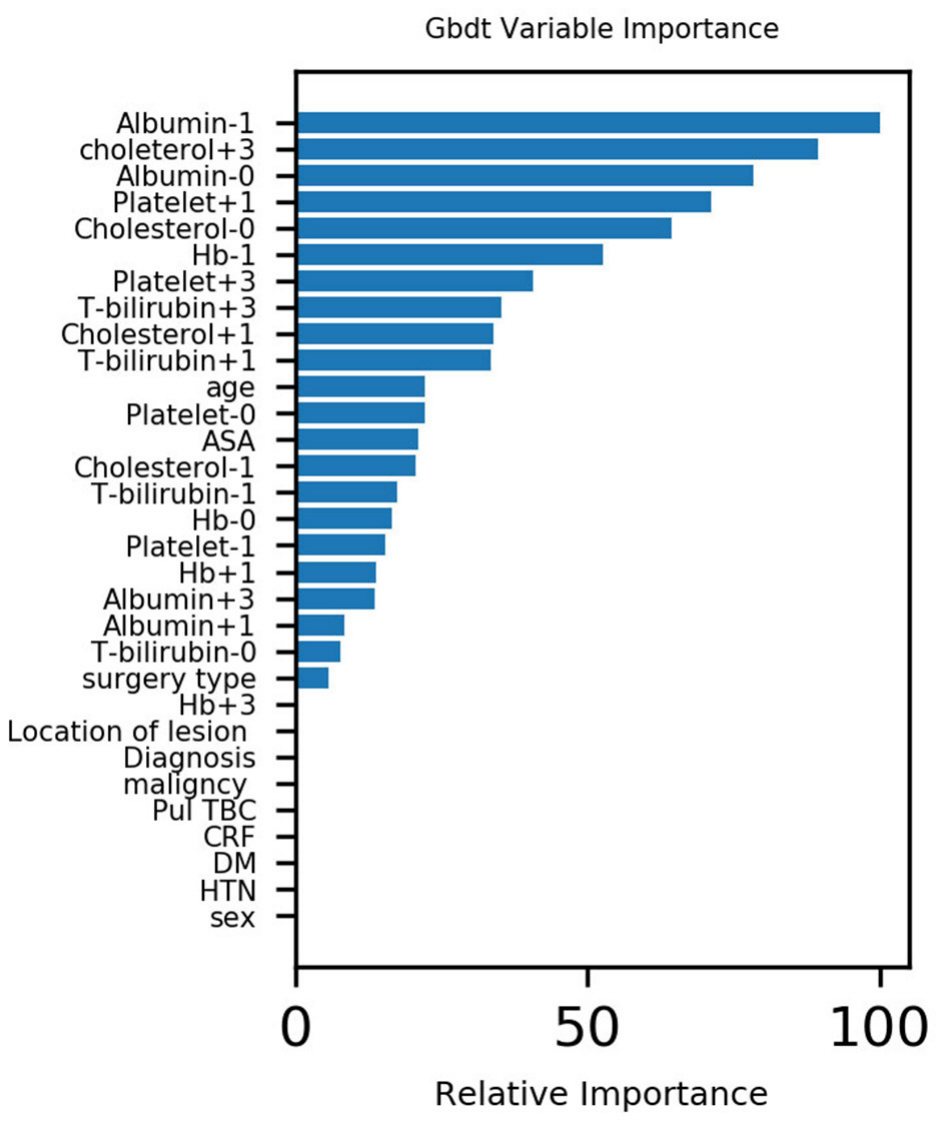
Variable importance of features included in machine learning algorithms for predicting pulmonary complications.

**Figure 2:**
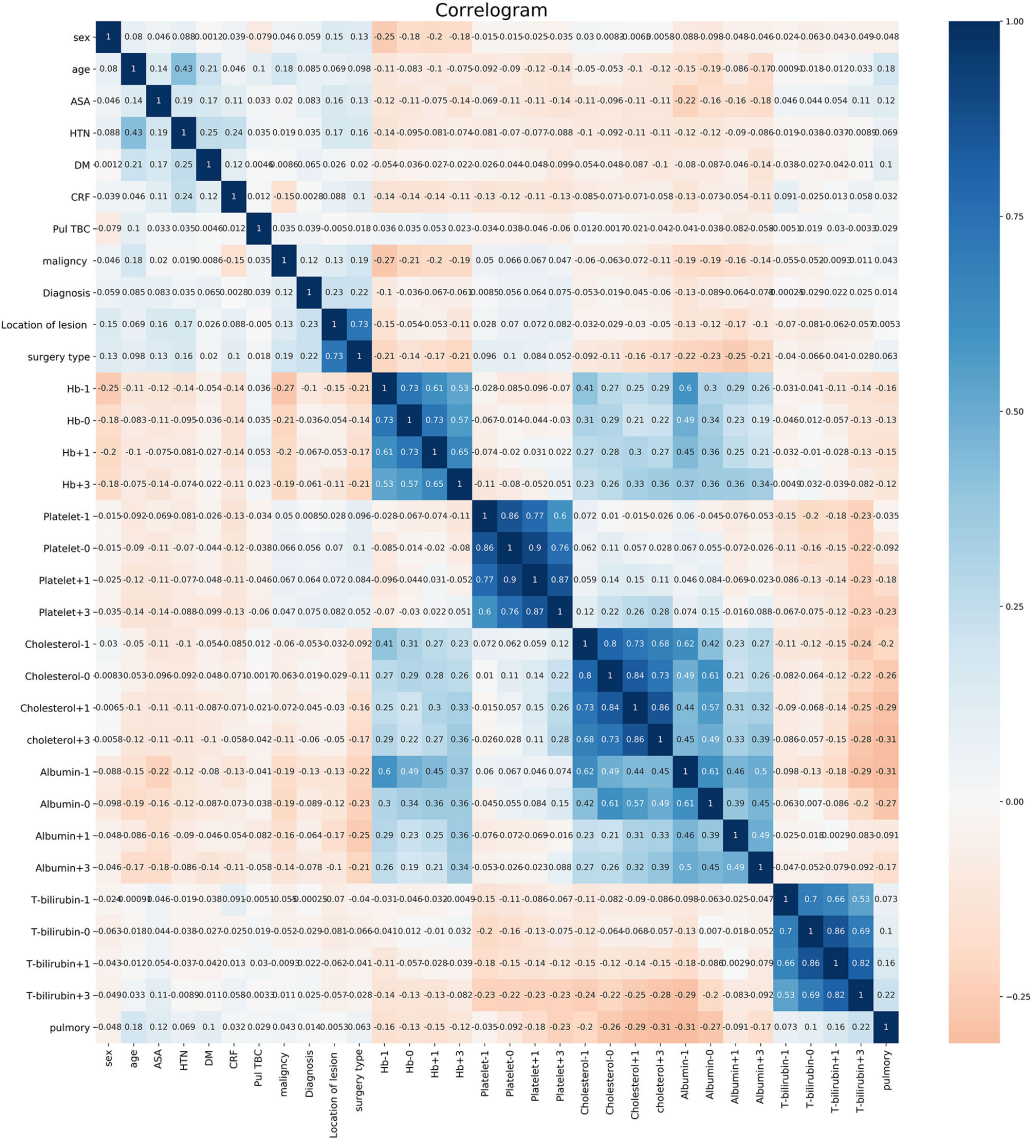
Correlation analysis of various factors.

The five most important variables for the postoperative weight were preoperative albumin, cholesterol on the 3rd day after surgery, albumin on the day of surgery, platelet count on the 1st day after surgery and cholesterol count on the 1st day after surgery for pulmonary complications. The correlation heat map showed that platelets, cholesterol, and albumin were negatively correlated with pulmonary complications (see Figures 1, 2).

**Figure 3:**
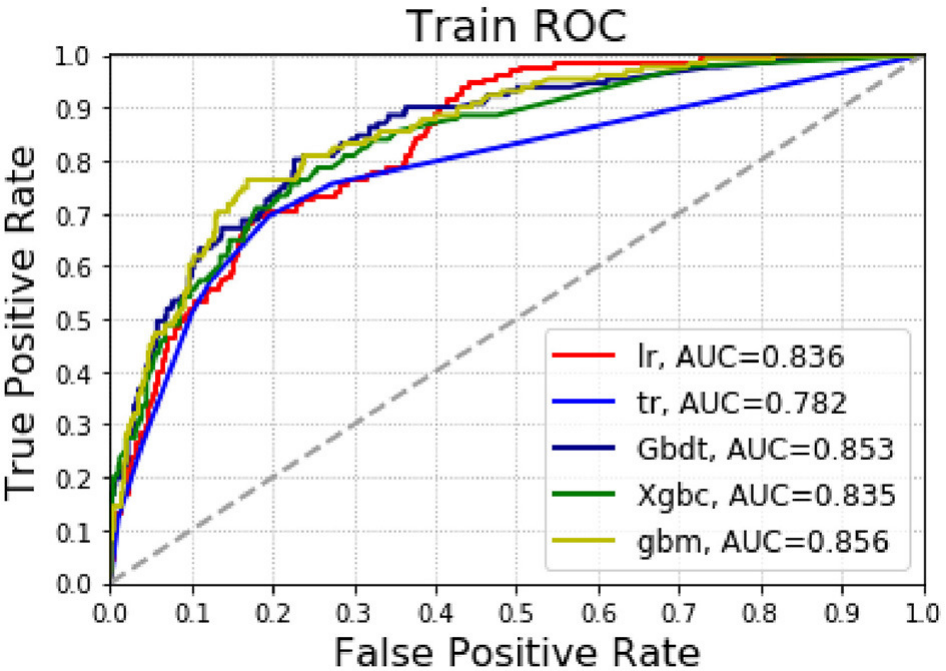
AUC for predicting pulmonary complications using various machine learning algorithms in the training group.

Supplementary Table 2 and Figure 3 present the results of the machine learning algorithm in the training group. The logistic regression model shows that AUC = 0.836, accuracy = 0.826 and precision = 0.625; Decision tree shows AUC = 0.782, accuracy = 0.821 and precision = 0.563; The GradientBoosting model shows AUC value = 0.853, accuracy = 0.824 and precision = 0.947; The Xgbc model shows AUC = 0.835, accuracy = 0.833 and precision = 0.897. The Gbm model shows AUC = 0.856, accuracy = 0.816 and precision = 0.929.

**Figure 4:**
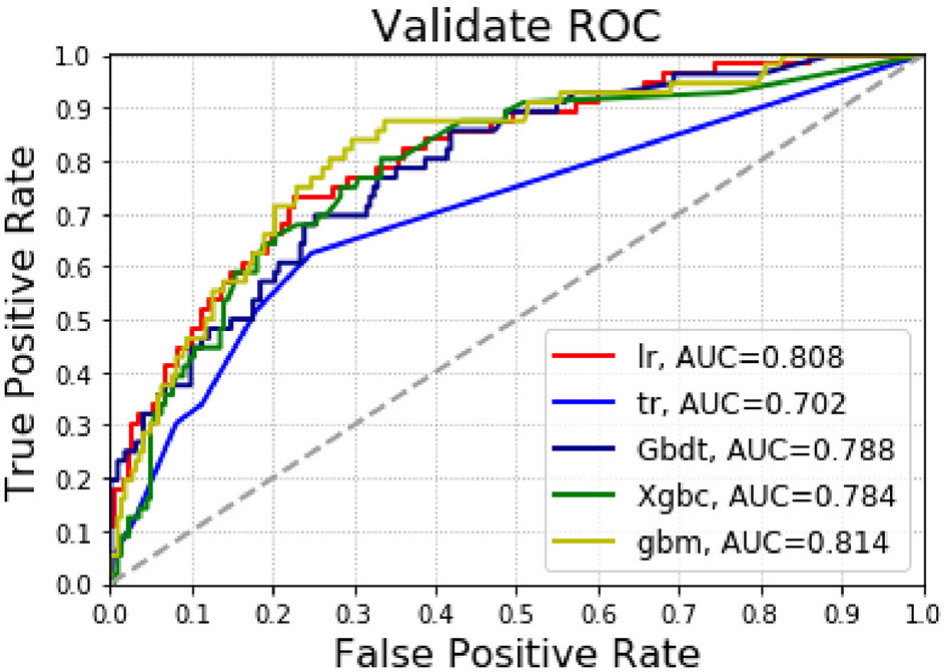
The AUC for predicting pulmonary complications using various machine learning algorithms in the test group.

Supplementary Table 2 and Figure 4 present the results of the machine learning algorithm in the test group. The logistic regression model shows AUC = 0.808, accuracy = 0.824 and precision = 0.621; Decision tree shows AUC = 0.702, accuracy = 0.795 and precision = 0.486; The GradientBoosting model shows AUC = 0.788, accuracy = 0.827 and precision = 1.000; The Xgbc model shows AUC = 0.784, accuracy =0.806 and precision = 0.583. The Gbm model shows AUC = 0.814, accuracy = 0.806 and precision = 0.750.

## Discussion

Postoperative pulmonary complications (PPCs) often occur after major surgery (18). Any PPC, even if it is “mild,” is associated with increased long-term hospitalization and hospital mortality (21). Thus, PPC predictions have the potential to optimize care for individual patients, normalize the use of scarce resources, and may even enrich research populations for testing PPC treatments' effects. Machine learning, using methods such as “unbiased cluster analysis” and biophenotypic analysis, likely improves PPC prediction models (22). The results of the present study show that machine learning algorithms can predict the postoperative pulmonary complications of patients with acute diffuse peritonitis.

Serum albumin levels represent a patient's nutritional status. Studies have shown that preoperative hypoproteinemia increases the incidence of postoperative abdominal complications (23). Other studies have shown that perioperative changes in serum albumin are predictors of lung complications in patients with lung cancer and laparoscopic gastrectomy (24, 25). Our results also indicate that changes in perioperative albumin are associated with changes in postoperative pulmonary complications. In addition, when ASA grade ≥ 3 and BMI is low, the incidence of pulmonary complications after early lung cancer radical surgery may increase (26). The present study's results also indicate that the ASA score is directly proportional to the occurrence of pulmonary complications.

Elevated levels of high-density lipoprotein cholesterol may be associated with decreased lung function in healthy male adolescents (27). Moreover, lower serum cholesterol levels are a poor prognostic factor in patients with severe community-acquired pneumonia (28). High cholesterol/high fat diet-induced hypercholesterolemia may result in lower respiratory inflammation associated with TLRs/NFκB pathway in C57BL/6J mice (29). Our study also supports this view, in that there is an inverse relationship between cholesterol and postoperative pulmonary complications.

Red blood cell distribution width and platelet count are biomarkers of pulmonary hypertension in patients with connective tissue disease (30). Also, platelet activating factor receptor regulates lung inflammation caused by colitis by NLRP3 inflammation (31). Platelets are factors in lung development in mice through Clec-2/podoplanin interactions (32). Our study also shows that perioperative platelet changes are a factor in the occurrence of PPCs.

Hemoglobin ≤ 100 g/L is an independent risk factor for postoperative pulmonary complications (33). Similarly, studies have suggested that serum albumin reduction on the 1st day after surgery can be a predictor of PPCs in patients with lung cancer after thoracoscopic anatomy (24). Our results also suggest that hemoglobin is an important contributor to postoperative complications, and that the two are inversely proportional.

This study has several limitations. First, because it was a retrospective study, the selected patient entry training and test datasets did not meet the predictions for the “future” cohort results. Thus, we needed to build a stable model to predict future postoperative pulmonary complications. In addition, the variables involved in this study may be insufficient. Future research should incorporate etiology and include more relevant influencing factors for analysis and research. Moreover, due to the limited predictive utility in our study, especially the ML algorithm's lower recall rate, there were several difficulties in applying the ML model in a clinical setting. However, with improved accuracy, this study's results are still reliable. Finally, the training sample size is still limited, as the cohort is from only one center. A multi-center prospective study is needed for training and validation in the future.

In sum, machine learning algorithms can predict the PPCs of patients with acute diffuse peritonitis. In future studies, specific machine learning models could be trained with a larger cohort of patients with acute diffuse peritonitis.

## Data Availability Statement

The original contributions presented in the study are included in the article/Supplementary Material, further inquiries can be directed to the corresponding author/s.

## Ethics Statement

Ethical review and approval was not required for the study on human participants in accordance with the local legislation and institutional requirements. Written informed consent for participation was not required for this study in accordance with the national legislation and the institutional requirements.

## Author Contributions

QX, DW, C-MZ, and J-JY were major contributors in writing the manuscript. All authors analyzed the data.

## Conflict of Interest

The authors declare that the research was conducted in the absence of any commercial or financial relationships that could be construed as a potential conflict of interest.

## Publisher's Note

All claims expressed in this article are solely those of the authors and do not necessarily represent those of their affiliated organizations, or those of the publisher, the editors and the reviewers. Any product that may be evaluated in this article, or claim that may be made by its manufacturer, is not guaranteed or endorsed by the publisher.

## References

[1] Sartelli M Catena F Balogh Z Bendinelli C Gupta S Kluger Y. Complicated intra-abdominal infections in a worldwide context: an observational prospective study (CIAOW Study) | NOVA. The University of Newcastle's Digital Repository. World J Emerg Surg. (2013) 8:1–7. 10.1186/1749-7922-8-1 23286785 PMC3538624

[2] Sartelli M. A focus on intra-abdominal infection. World J Emerg Surg. (2010) 5:9. 10.1186/1749-7922-5-9 20302628 PMC2848006

[3] Lee SH Lee JY Hong TH Kim BO Lee YJ Lee JG. Severe persistent hypocholesterolemia after emergency gastrointestinal surgery predicts in-hospital mortality in critically ill patients with diffuse peritonitis. PLoS ONE. (2018) 13:e0200187. 10.1371/journal.pone.0200187 29979773 PMC6034864

[4] Smetana GW Lawrence VA Cornell JE. Preoperative pulmonary risk stratification for noncardiothoracic surgery: systematic review for the American College of Physicians. Ann Intern Med. (2006) 144:581–95. 10.7326/0003-4819-144-8-200604180-00009 16618956

[5] Overend TJ Anderson CM Lucy SD Bhatia C Jonsson BI Timmermans C. The effect of incentive spirometry on postoperative pulmonary complications: a systematic review. Chest. (2001) 120:971–8. 10.1378/chest.120.3.971 11555536

[6] Canet J Gallart L Gomar C Paluzie G Vallès J Castillo J. Prediction of postoperative pulmonary complications in a population-based surgical cohort. Anesthesiology. (2010) 113:1338–50. 10.1097/ALN.0b013e3181fc6e0a 21045639

[7] Weingarten TN Kor DJ Gali B Sprung J. Predicting postoperative pulmonary complications in high-risk populations. Curr Opin Anaesthesiol. (2013) 26:116–25. 10.1097/ACO.0b013e32835e21d2 23407151 PMC4844023

[8] Neto AS Bos LD Campos P Hemmes S Schultz MJ. Association between pre-operative biological phenotypes and postoperative pulmonary complications: an unbiased cluster analysis. Eur J Anaesthesiol. (2018) 35:1. 10.1097/EJA.0000000000000846 29957706

[9] Shameer K Johnson KW Glicksberg BS Dudley JT Sengupta PP. Machine learning in cardiovascular medicine: are we there yet? Heart. (2018) 104:1156–64. 10.1136/heartjnl-2017-311198 29352006

[10] Bibault JE Giraud P. Burgun Anita. Big Data and machine learning in radiation oncology: state of the art and future prospects. Cancer Lett. (2016) 382:110–7. 10.1016/j.canlet.2016.05.033 27241666

[11] Zhou CM Xue Q Wang Y Tong J Ji M Yang JJ. Machine learning to predict the cancer-specific mortality of patients with primary non-metastatic invasive breast cancer. Surg Today Surg Today. (2021) 51:756–63. 10.1007/s00595-020-02170-9 33104877

[12] Zhou ZH. Ensemble Methods - Foundations and Algorithms. Boca Raton, FL: Taylor & Francis Group (2012) p. 77–9. 10.1201/b12207

[13] Fei Y Gao K Li WQ. Artificial neural network algorithm model as powerful tool to predict acute lung injury following to severe acute pancreatitis. Pancreatology. (2018) 18:892–9. 10.1016/j.pan.2018.09.007 30268673

[14] Nijbroek SG Schultz MJ Hemmes SNT. Prediction of postoperative pulmonary complications. Curr Opin Anaesthesiol. (2019) 32:443–51. 10.1097/ACO.0000000000000730 30893115

[15] Christodoulou E Ma J Collins GS Steyerberg EW Verbakel JY Van CB. systematic review shows no performance benefit of machine learning over logistic regression for clinical prediction models. J Clin Epidemiol. (2019) 110:12–22. 10.1016/j.jclinepi.2019.02.004 30763612

[16] Praagman J. Classification and regression trees: Leo Breiman, Jerome H. Friedman, Richard A. Olshen and Charles J. Stone. The Wadsworth Statistics/Probability Series, Wadsworth, Belmont, 1984, x + 358 pages. Eur J Operation Res. (1985) 19:144. 10.1016/0377-2217(85)90321-2

[17] Liao Z Yong H Yue X Lu H Ying J. *In silico* prediction of gamma-aminobutyric acid type-a receptors using novel machine-learning-based SVM and GBDT approaches. Biomed Res Int. (2016) 2016:2375268. 10.1155/2016/2375268 27579307 PMC4992803

[18] Zhang J Mucs D Norinder U Svensson F. LightGBM: an effective and scalable algorithm for prediction of chemical toxicity-application to the Tox21 and mutagenicity data sets. J Chem Inf Model. (2019) 59:4150–8. 10.1021/acs.jcim.9b00633 31560206

[19] Zhou CM Wang Y Ye HT Yan S Ji M Liu P. Machine learning predicts lymph node metastasis of poorly differentiated-type intramucosal gastric cancer. Sci Rep. (2021) 11:1300. 10.1038/s41598-020-80582-w 33446730 PMC7809018

[20] Zhou C Hu J Wang Y Ji MH Tong J Yang JJ. A machine learning-based predictor for the identification of the recurrence of patients with gastric cancer after operation. Sci Rep. (2021) 11:1571. 10.1038/s41598-021-81188-6 33452440 PMC7810757

[21] LAS VEGAS Investigators. Epidemiology, practice of ventilation and outcome for patients at increased risk of postoperative pulmonary complications: LAS VEGAS - an observational study in 29 countries. Eur J Anaesthesiol. (2017) 34:492–507. 10.1097/EJA.0000000000000646 28633157 PMC5502122

[22] Serpa NA Hemmes SN Barbas CS Beiderlinden M Fernandez-Bustamante A Futier E. Incidence of mortality and morbidity related to postoperative lung injury in patients who have undergone abdominal or thoracic surgery: a systematic review and meta-analysis. Lancet Respir Med. (2014) 2:1007–15. 10.1016/S2213-2600(14)70228-0 25466352

[23] Rock P Rich PB. Postoperative pulmonary complications. Curr Opin Anaesthesiol. (2003) 16:123–31. 10.1097/00001503-200304000-00004 17021450

[24] Li P Li J Lai Y Wang Y Wang X Su J. Perioperative changes of serum albumin are a predictor of postoperative pulmonary complications in lung cancer patients: a retrospective cohort study. J Thorac Dis. (2018) 10:5755–63. 10.21037/jtd.2018.09.113 30505483 PMC6236178

[25] Chen Y Wu G Wang R Chen J. Preoperative albumin level serves as a predictor for postoperative pulmonary complications following elective laparoscopic gastrectomy. Curr Pharm Des. (2018) 24:3250–5. 10.2174/1381612824666180713104307 30003856

[26] Im Y Park HY Shin S Shin SH Lee H Ahn JH. Prevalence of and risk factors for pulmonary complications after curative resection in otherwise healthy elderly patients with early stage lung cancer. Respir Res. (2019) 20:136. 10.1186/s12931-019-1087-x 31272446 PMC6610954

[27] Park JH Mun S Choi DP Lee JY Kim HC. Association between high-density lipoprotein cholesterol level and pulmonary function in healthy Korean adolescents: the JS high school study. BMC Pulm Med. (2017) 17:190. 10.1186/s12890-017-0548-6 29228928 PMC5725943

[28] Chien YF Chen CY Hsu CL Chen KY Yu CJ. Decreased serum level of lipoprotein cholesterol is a poor prognostic factor for patients with severe community-acquired pneumonia that required intensive care unit admission. J Crit Care. (2015) 30:506–10. 10.1016/j.jcrc.2015.01.001 25702844

[29] Fang Y Wang S Zhu T Zhang Y Lian X. Atherogenic high cholesterol/high fat diet induces TLRs-associated pulmonary inflammation in C57BL/6J mice. Inflamm Res. (2017) 66:39–47. 10.1007/s00011-016-0990-6 27653962

[30] Bellan M Giubertoni A Piccinino C Dimagli A Grimoldi F Sguazzotti M. Red cell distribution width and platelet count as biomarkers of pulmonary arterial hypertension in patients with connective tissue disorders. Dis Markers. (2019) 2019:4981982. 10.1155/2019/4981982 31275447 PMC6589198

[31] Liu G Mateer SW Hsu A Goggins BJ Tay H Mathe A. Platelet activating factor receptor regulates colitis-induced pulmonary inflammation through the NLRP3 inflammasome. Mucosal Immunol. (2019) 12:862–73. 10.1038/s41385-019-0163-3 30976089

[32] Tsukiji N Inoue O Morimoto M Tatsumi N Nagatomo H Ueta K. Platelets play an essential role in murine lung development through Clec-2/podoplanin interaction. Blood. (2018) 132:1167–79. 10.1182/blood-2017-12-823369 29853539

[33] Jiang GQ Bai DS Chen P Fan J Tan JW Peng MH. Starting hemoglobin value predicts early phase prognosis after liver transplantation. Transplant Proc. (2011) 43:1669–73. 10.1016/j.transproceed.2010.12.067 21693255

