# Supplementary material for: Developing Machine Learning Algorithms to Predict Pulmonary Complications After Emergency Gastrointestinal Surgery
Source: Front Med (Lausanne). 2021 Aug 2;8:655686. doi: 10.3389/fmed.2021.655686 (PMC8365303; doi:10.3389/fmed.2021.655686)
Supplement: Supplementary file 1 [file Table_1.DOC]

**Supplementary Table 1** Baseline data.

| PULMORY | No | Yes | P-value |
| --- | --- | --- | --- |
| N | 739 | 187 |  |
| AGE (year) | 58.2 ± 16.1 | 65.6 ± 14.5 | <0.001 |
| HB-1, g/dL | 12.2 ± 2.4 | 11.2 ± 2.3 | <0.001 |
| HB+0, g/dL | 11.6 ± 2.1 | 10.9 ± 2.3 | <0.001 |
| HB+.1, g/dL | 10.7 ± 2.0 | 9.9 ± 1.8 | <0.001 |
| HB+3, g/dL | 10.2 ± 1.7 | 9.7 ± 1.5 | <0.001 |
| PLATELET-1,10^3^/μL | 271.6 ± 128.1 | 260.5 ± 146.1 | 0.123 |
| PLATELET+0,10^3^/μL | 247.4 ± 122.8 | 218.8 ± 130.0 | <0.001 |
| PLATELET+1,10^3^/μL | 225.4 ± 110.8 | 176.4 ± 108.7 | <0.001 |
| PLATELET+3,10^3^/μL | 224.1 ± 111.6 | 157.6 ± 110.9 | <0.001 |
| CHOLESTEROL-1, mg/dL | 143.6 ± 49.5 | 118.5 ± 43.9 | <0.001 |
| CHOLESTEROL+0, mg/dL | 109.1 ± 47.7 | 77.9 ± 37.7 | <0.001 |
| CHOLESTEROL+1, mg/dL | 89.5 ± 38.3 | 62.3 ± 25.1 | <0.001 |
| CHOLETEROL+3, mg/dL | 101.4 ± 36.9 | 73.2 ± 27.4 | <0.001 |
| ALBUMIN-1, g/dL | 3.5 ± 0.7 | 2.9 ± 0.6 | <0.001 |
| ALBUMIN+0, g/dL | 2.7 ± 0.7 | 2.3 ± 0.6 | <0.001 |
| ALBUMIN+1, g/dL | 2.8 ± 0.4 | 2.7 ± 0.4 | 0.011 |
| ALBUMIN+3, g/dL | 2.9 ± 0.4 | 2.8 ± 0.3 | <0.001 |
| T-BILIRUBIN-1, mg/dL | 1.0 ± 1.3 | 1.2 ± 1.3 | 0.027 |
| T-BILIRUBIN+0, mg/dL | 1.0 ± 0.9 | 1.3 ± 1.3 | 0.269 |
| T-BILIRUBIN+1, mg/dL | 1.1 ± 1.0 | 1.5 ± 1.6 | 0.004 |
| T-BILIRUBIN+3, mg/dL | 1.0 ± 1.2 | 1.8 ± 2.0 | <0.001 |
| SEX |  |  | 0.142 |
| Male | 455 (61.6%) | 126 (67.4%) |  |
| Female | 284 (38.4%) | 61 (32.6%) |  |
| ASA |  |  | <0.001 |
| 1 | 266 (36.0%) | 65 (34.8%) |  |
| 2 | 245 (33.2%) | 35 (18.7%) |  |
| 3 | 189 (25.6%) | 56 (29.9%) |  |
| 4 | 38 ( 5.1%) | 29 (15.5%) |  |
| 5 | 1 ( 0.1%) | 2 ( 1.1%) |  |
| HTN |  |  | 0.037 |
| No | 506 (68.5%) | 113 (60.4%) |  |
| Yes | 233 (31.5%) | 74 (39.6%) |  |
| DM |  |  | 0.001 |
| No | 648 (87.7%) | 147 (78.6%) |  |
| Yes | 91 (12.3%) | 40 (21.4%) |  |
| CRF |  |  | 0.332 |
| No | 701 (94.9%) | 174 (93.0%) |  |
| Yes | 38 ( 5.1%) | 13 ( 7.0%) |  |
| PUL.TBC |  |  | 0.376 |
| No | 693 (93.8%) | 172 (92.0%) |  |
| Yes | 46 ( 6.2%) | 15 ( 8.0%) |  |
| MALIGNCY |  |  | 0.187 |
| No | 360 (48.7%) | 81 (43.3%) |  |
| Yes | 379 (51.3%) | 106 (56.7%) |  |
| DIAGNOSIS |  |  | 0.004 |
| Perforation | 525 (71.0%) | 143 (76.5%) |  |
| Strangulation | 156 (21.1%) | 20 (10.7%) |  |
| Anastomotic leakage | 22 ( 3.0%) | 9 ( 4.8%) |  |
| Acute mesenteric ischemia | 36 ( 4.9%) | 15 ( 8.0%) |  |
| LOCATION.OF.LESION |  |  | 0.937 |
| Stomach | 114 (15.4%) | 27 (14.4%) |  |
| Duodenum | 47 ( 6.4%) | 14 ( 7.5%) |  |
| Jejunum and ileum | 254 (34.4%) | 63 (33.7%) |  |
| Colon and rectum | 324 (43.8%) | 83 (44.4%) |  |
| SURGERY.TYPE |  |  | 0.023 |
| Primary repair | 191 (25.8%) | 45 (24.1%) |  |
| Small bowel resection with anastomosis | 40 ( 5.4%) | 10 ( 5.3%) |  |
| Ileo- or jejunostomy | 174 (23.5%) | 39 (20.9%) |  |
| Hartmann`s procedures or colostomy | 118 (16.0%) | 17 ( 9.1%) |  |
| Colon resection with anastomosis | 112 (15.2%) | 34 (18.2%) |  |
| Gastrectomy (subtotal or total) | 104 (14.1%) | 42 (22.5%) |  |

Results in table: Mean+SD/N(%)

P value: if it is a continuous variable, it is obtained by Wilcoxon rank-sum test rank sum test, if the counting variable has theoretical number < 10, it is obtained by Fisher exact probability test

Hb, Hemoglobin; HTN, Hypertension; DM, diabetes mellitus; CRF, chronic renal failure; ASA, American Society of Anesthesiologists physical status classification; CRP, C-reactive protein; PUL.TBC, Pulmonary tuberculosis;-1, preoperative,+0, on the day of surgery; +1, on the first day after operation; +3, on the 3rd day after operation.
