# Supplementary material for: Developing Machine Learning Algorithms to Predict Pulmonary Complications After Emergency Gastrointestinal Surgery
Source: Front Med (Lausanne). 2021 Aug 2;8:655686. doi: 10.3389/fmed.2021.655686 (PMC8365303; doi:10.3389/fmed.2021.655686)
Supplement: Supplementary file 2 [file Table_2.doc]

**Supplementary Table 2** Forecast Results for Training and Test Groups

|  | Training | | | | | Test | | | | |
| --- | --- | --- | --- | --- | --- | --- | --- | --- | --- | --- |
|  | Accuracy | Precision | Recall | F1_score | AUC | Accuracy | Precision | Recall | F1_score | AUC |
| Logistic | 0.826 | 0.625 | 0.344 | 0.443 | 0.836 | 0.824 | 0.621 | 0.321 | 0.424 | 0.807 |
| DecisionTree | 0.821 | 0.563 | 0.511 | 0.536 | 0.782 | 0.795 | 0.486 | 0.304 | 0.374 | 0.702 |
| GradientBoosting | 0.824 | 0.947 | 0.137 | 0.240 | 0.853 | 0.827 | 1.000 | 0.143 | 0.250 | 0.788 |
| Xgbc | 0.833 | 0.897 | 0.198 | 0.325 | 0.835 | 0.806 | 0.583 | 0.125 | 0.206 | 0.784 |
| Lightgbm | 0.816 | 0.929 | 0.099 | 0.179 | 0.856 | 0.806 | 0.750 | 0.054 | 0.100 | 0.814 |
