# Supplementary material for: Developing Machine Learning Algorithms to Predict Pulmonary Complications After Emergency Gastrointestinal Surgery
Source: Front Med (Lausanne). 2021 Aug 2;8:655686. doi: 10.3389/fmed.2021.655686 (PMC8365303; doi:10.3389/fmed.2021.655686)
Supplement: Supplementary file 3 [file Data_Sheet_1.PDF]

In [16]:

```
# 分模型进行训练、评分

lr = LogisticRegression(penalty='l2',tol=0.0000001,C=10,fit_intercept=True,intercept_scaling=1,
max_iter=100,multi_class='ovr',verbose=0,warm_start=False,n_jobs=1)  # 逻辑回归模型
lr_result = model_fit_score(lr, X_train, y_train)
model_print(lr_result, "LogisticRegression - Train")

tr = DecisionTreeClassifier(splitter='best', max_depth=3, min_samples_split=60,min_samples_leaf
=20, min_weight_fraction_leaf=0.001,  random_state=1)  # 决策树模型
tr_result = model_fit_score(tr, X_train, y_train)
model_print(tr_result, "DecisionTreeClassifier - Train")

Gbdt = GradientBoostingClassifier(learning_rate=0.1,n_estimators=20,max_depth=2, max_features=3,
min_samples_split=20,min_samples_leaf=3,random_state =1)#CBDT
Gbdt_result = model_fit_score(Gbdt, X_train, y_train)
model_print(Gbdt_result, "GradientBoostingClassifier - Train")

Xgbc = XGBClassifier(learning_rate=0.1,n_estimators=10,  max_depth=2,  min_child_weight = 1,gamm
a=0.1,scale_pos_weight=1) #Xgbc
Xgbc_result = model_fit_score(Xgbc, X_train, y_train)
model_print(Xgbc_result, "XGBClassifier - Train")

gbm = lgb.LGBMClassifier(learning_rate=0.1, n_estimators=30, lambda_l1=0.01 ,lambda_l2= 10 ,max_
depth=2, bagging_fraction = 0.8,feature_fraction = 0.5) #lgb
gbm_result = model_fit_score(gbm, X_train, y_train)
model_print(gbm_result, "LGBMClassifier - Train")
```

```
[LogisticRegression - Train] accuracy_score: 0.826, preci_score: 0.625, recall_sco
re: 0.344, f1_score: 0.443, auc: 0.836,
 mse: 0.174, zero_one_loss_fraction: 0.174, zero_one_loss_num: 113.000, cv_score:
0.607
confusion_matrix:
 [[490  27]
 [ 86  45]]


[DecisionTreeClassifier - Train] accuracy_score: 0.821, preci_score: 0.563, recall
_score: 0.511, f1_score: 0.536, auc: 0.782,
 mse: 0.179, zero_one_loss_fraction: 0.179, zero_one_loss_num: 116.000, cv_score:
0.564
confusion_matrix:
 [[465  52]
 [ 64  67]]


[GradientBoostingClassifier - Train] accuracy_score: 0.824, preci_score: 0.947, re
call_score: 0.137, f1_score: 0.240, auc: 0.853,
 mse: 0.176, zero_one_loss_fraction: 0.176, zero_one_loss_num: 114.000, cv_score:
0.520
confusion_matrix:
 [[516   1]
 [113  18]]



C:\Anaconda3\lib\site-packages\xgboost\sklearn.py:888: UserWarning: The use of lab
el encoder in XGBClassifier is deprecated and will be removed in a future release.
To remove this warning, do the following: 1) Pass option use_label_encoder=False w
hen constructing XGBClassifier object; and 2) Encode your labels (y) as integers s
tarting with 0, i.e. 0, 1, 2, ..., [num_class - 1].
  warnings.warn(label_encoder_deprecation_msg, UserWarning)

[22:45:37] WARNING: C:/Users/Administrator/workspace/xgboost-win64_release_1.3.0/s
rc/learner.cc:1061: Starting in XGBoost 1.3.0, the default evaluation metric used
with the objective 'binary:logistic' was changed from 'error' to 'logloss'. Explic
itly set eval_metric if you'd like to restore the old behavior.
[22:45:37] WARNING: C:/Users/Administrator/workspace/xgboost-win64_release_1.3.0/s
rc/learner.cc:1061: Starting in XGBoost 1.3.0, the default evaluation metric used
with the objective 'binary:logistic' was changed from 'error' to 'logloss'. Explic
itly set eval_metric if you'd like to restore the old behavior.
[22:45:37] WARNING: C:/Users/Administrator/workspace/xgboost-win64_release_1.3.0/s
rc/learner.cc:1061: Starting in XGBoost 1.3.0, the default evaluation metric used
with the objective 'binary:logistic' was changed from 'error' to 'logloss'. Explic
itly set eval_metric if you'd like to restore the old behavior.
[22:45:37] WARNING: C:/Users/Administrator/workspace/xgboost-win64_release_1.3.0/s
rc/learner.cc:1061: Starting in XGBoost 1.3.0, the default evaluation metric used
with the objective 'binary:logistic' was changed from 'error' to 'logloss'. Explic
itly set eval_metric if you'd like to restore the old behavior.
[22:45:37] WARNING: C:/Users/Administrator/workspace/xgboost-win64_release_1.3.0/s
rc/learner.cc:1061: Starting in XGBoost 1.3.0, the default evaluation metric used
with the objective 'binary:logistic' was changed from 'error' to 'logloss'. Explic
itly set eval_metric if you'd like to restore the old behavior.
```

```
C:\Anaconda3\lib\site-packages\xgboost\sklearn.py:888: UserWarning: The use of lab
el encoder in XGBClassifier is deprecated and will be removed in a future release.
To remove this warning, do the following: 1) Pass option use_label_encoder=False w
hen constructing XGBClassifier object; and 2) Encode your labels (y) as integers s
tarting with 0, i.e. 0, 1, 2, ..., [num_class - 1].
  warnings.warn(label_encoder_deprecation_msg, UserWarning)
C:\Anaconda3\lib\site-packages\xgboost\sklearn.py:888: UserWarning: The use of lab
el encoder in XGBClassifier is deprecated and will be removed in a future release.
To remove this warning, do the following: 1) Pass option use_label_encoder=False w
hen constructing XGBClassifier object; and 2) Encode your labels (y) as integers s
tarting with 0, i.e. 0, 1, 2, ..., [num_class - 1].
  warnings.warn(label_encoder_deprecation_msg, UserWarning)
C:\Anaconda3\lib\site-packages\xgboost\sklearn.py:888: UserWarning: The use of lab
el encoder in XGBClassifier is deprecated and will be removed in a future release.
To remove this warning, do the following: 1) Pass option use_label_encoder=False w
hen constructing XGBClassifier object; and 2) Encode your labels (y) as integers s
tarting with 0, i.e. 0, 1, 2, ..., [num_class - 1].
  warnings.warn(label_encoder_deprecation_msg, UserWarning)
C:\Anaconda3\lib\site-packages\xgboost\sklearn.py:888: UserWarning: The use of lab
el encoder in XGBClassifier is deprecated and will be removed in a future release.
To remove this warning, do the following: 1) Pass option use_label_encoder=False w
hen constructing XGBClassifier object; and 2) Encode your labels (y) as integers s
tarting with 0, i.e. 0, 1, 2, ..., [num_class - 1].
  warnings.warn(label_encoder_deprecation_msg, UserWarning)
C:\Anaconda3\lib\site-packages\xgboost\sklearn.py:888: UserWarning: The use of lab
el encoder in XGBClassifier is deprecated and will be removed in a future release.
To remove this warning, do the following: 1) Pass option use_label_encoder=False w
hen constructing XGBClassifier object; and 2) Encode your labels (y) as integers s
tarting with 0, i.e. 0, 1, 2, ..., [num_class - 1].
  warnings.warn(label_encoder_deprecation_msg, UserWarning)
```

```
[22:45:37] WARNING: C:/Users/Administrator/workspace/xgboost-win64_release_1.3.0/s
rc/learner.cc:1061: Starting in XGBoost 1.3.0, the default evaluation metric used
with the objective 'binary:logistic' was changed from 'error' to 'logloss'. Explic
itly set eval_metric if you'd like to restore the old behavior.
[XGBClassifier - Train] accuracy_score: 0.833, preci_score: 0.897, recall_score:
0.198, f1_score: 0.325, auc: 0.835,
 mse: 0.167, zero_one_loss_fraction: 0.167, zero_one_loss_num: 108.000, cv_score:
0.515
confusion_matrix:
 [[514   3]
 [105  26]]


[LightGBM] [Warning] feature_fraction is set=0.5, colsample_bytree=1.0 will be ign
ored. Current value: feature_fraction=0.5
[LightGBM] [Warning] lambda_l1 is set=0.01, reg_alpha=0.0 will be ignored. Current
value: lambda_l1=0.01
[LightGBM] [Warning] bagging_fraction is set=0.8, subsample=1.0 will be ignored. C
urrent value: bagging_fraction=0.8
[LightGBM] [Warning] lambda_l2 is set=10, reg_lambda=0.0 will be ignored. Current
value: lambda_l2=10
[LightGBM] [Warning] feature_fraction is set=0.5, colsample_bytree=1.0 will be ign
ored. Current value: feature_fraction=0.5
[LightGBM] [Warning] lambda_l1 is set=0.01, reg_alpha=0.0 will be ignored. Current
value: lambda_l1=0.01
[LightGBM] [Warning] bagging_fraction is set=0.8, subsample=1.0 will be ignored. C
urrent value: bagging_fraction=0.8
[LightGBM] [Warning] lambda_l2 is set=10, reg_lambda=0.0 will be ignored. Current
value: lambda_l2=10
[LightGBM] [Warning] feature_fraction is set=0.5, colsample_bytree=1.0 will be ign
ored. Current value: feature_fraction=0.5
[LightGBM] [Warning] lambda_l1 is set=0.01, reg_alpha=0.0 will be ignored. Current
value: lambda_l1=0.01
[LightGBM] [Warning] bagging_fraction is set=0.8, subsample=1.0 will be ignored. C
urrent value: bagging_fraction=0.8
[LightGBM] [Warning] lambda_l2 is set=10, reg_lambda=0.0 will be ignored. Current
value: lambda_l2=10
[LightGBM] [Warning] feature_fraction is set=0.5, colsample_bytree=1.0 will be ign
ored. Current value: feature_fraction=0.5
[LightGBM] [Warning] lambda_l1 is set=0.01, reg_alpha=0.0 will be ignored. Current
value: lambda_l1=0.01
[LightGBM] [Warning] bagging_fraction is set=0.8, subsample=1.0 will be ignored. C
urrent value: bagging_fraction=0.8
[LightGBM] [Warning] lambda_l2 is set=10, reg_lambda=0.0 will be ignored. Current
value: lambda_l2=10
[LightGBM] [Warning] feature_fraction is set=0.5, colsample_bytree=1.0 will be ign
ored. Current value: feature_fraction=0.5
[LightGBM] [Warning] lambda_l1 is set=0.01, reg_alpha=0.0 will be ignored. Current
value: lambda_l1=0.01
[LightGBM] [Warning] bagging_fraction is set=0.8, subsample=1.0 will be ignored. C
urrent value: bagging_fraction=0.8
[LightGBM] [Warning] lambda_l2 is set=10, reg_lambda=0.0 will be ignored. Current
value: lambda_l2=10
[LightGBM] [Warning] feature_fraction is set=0.5, colsample_bytree=1.0 will be ign
ored. Current value: feature_fraction=0.5
[LightGBM] [Warning] lambda_l1 is set=0.01, reg_alpha=0.0 will be ignored. Current
value: lambda_l1=0.01
[LightGBM] [Warning] bagging_fraction is set=0.8, subsample=1.0 will be ignored. C
urrent value: bagging_fraction=0.8
[LightGBM] [Warning] lambda_l2 is set=10, reg_lambda=0.0 will be ignored. Current
value: lambda_l2=10
```

[LGBMClassifier - Train] accuracy_score: 0.816, preci_score: 0.929, recall_score: 0.099, f1_score: 0.179, auc: 0.856,
 mse: 0.184, zero_one_loss_fraction: 0.184, zero_one_loss_num: 119.000, cv_score: 0.504
confusion_matrix:
```
 [[516   1]
 [118  13]]
```

In [17]:

```
# 训练集：画roc/auc图
plt_roc_auc([
    (lr_result, 'lr', ),
    (tr_result, 'tr', ),
    (Gbdt_result, 'Gbdt', ),
    (Xgbc_result, 'Xgbc', ),
    (gbm_result, 'gbm', ),
], 'Train ROC')
```

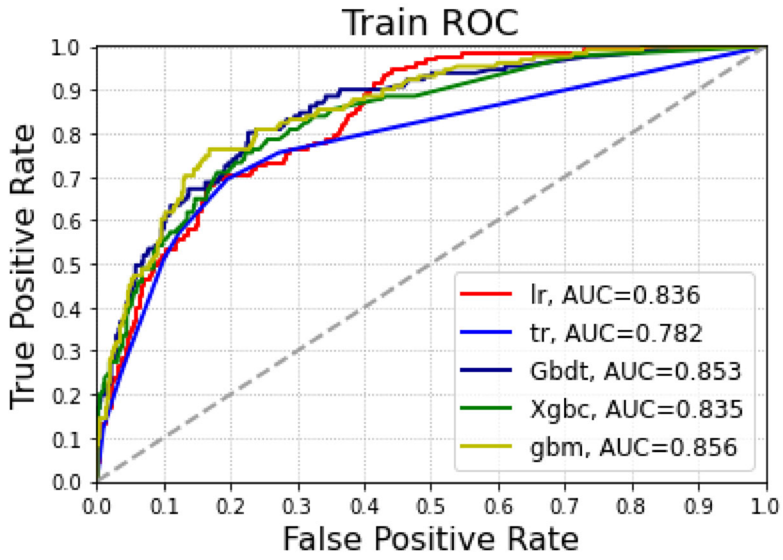

<Figure size 4800x9000 with 0 Axes>

In [18]:

```
# 分模型进行测试、评分（注：用训练集训练后的同个模型 进行测试集的测试）

lr_test_result = model_score(lr, X_test, y_test)
model_print(lr_test_result, "LogisticRegression - Test")

tr_test_result = model_score(tr, X_test, y_test)
model_print(tr_test_result, "DecisionTreeClassifier - Test")



Gbdt_test_result = model_score(Gbdt, X_test, y_test)
model_print(Gbdt_test_result, "GradientBoostingClassifier - Test")

Xgbc_test_result = model_score(Xgbc, X_test, y_test)
model_print(Xgbc_test_result, "XGBClassifier - Test")

gbm_test_result = model_score(gbm, X_test, y_test)
model_print(gbm_test_result, "LGBMClassifier - Test")
```

```
[LogisticRegression - Test] accuracy_score: 0.824, preci_score: 0.621, recall_scor
e: 0.321, f1_score: 0.424, auc: 0.807,
 mse: 0.176, zero_one_loss_fraction: 0.176, zero_one_loss_num: 49.000, cv_score:
0.611
confusion_matrix:
 [[211  11]
 [ 38  18]]


[DecisionTreeClassifier - Test] accuracy_score: 0.795, preci_score: 0.486, recall_
score: 0.304, f1_score: 0.374, auc: 0.702,
 mse: 0.205, zero_one_loss_fraction: 0.205, zero_one_loss_num: 57.000, cv_score:
0.660
confusion_matrix:
 [[204  18]
 [ 39  17]]


[GradientBoostingClassifier - Test] accuracy_score: 0.827, preci_score: 1.000, rec
all_score: 0.143, f1_score: 0.250, auc: 0.788,
 mse: 0.173, zero_one_loss_fraction: 0.173, zero_one_loss_num: 48.000, cv_score:
0.556
confusion_matrix:
 [[222   0]
 [ 48   8]]



C:\Anaconda3\lib\site-packages\xgboost\sklearn.py:888: UserWarning: The use of lab
el encoder in XGBClassifier is deprecated and will be removed in a future release.
To remove this warning, do the following: 1) Pass option use_label_encoder=False w
hen constructing XGBClassifier object; and 2) Encode your labels (y) as integers s
tarting with 0, i.e. 0, 1, 2, ..., [num_class - 1].
  warnings.warn(label_encoder_deprecation_msg, UserWarning)
C:\Anaconda3\lib\site-packages\xgboost\sklearn.py:888: UserWarning: The use of lab
el encoder in XGBClassifier is deprecated and will be removed in a future release.
To remove this warning, do the following: 1) Pass option use_label_encoder=False w
hen constructing XGBClassifier object; and 2) Encode your labels (y) as integers s
tarting with 0, i.e. 0, 1, 2, ..., [num_class - 1].
  warnings.warn(label_encoder_deprecation_msg, UserWarning)
C:\Anaconda3\lib\site-packages\xgboost\sklearn.py:888: UserWarning: The use of lab
el encoder in XGBClassifier is deprecated and will be removed in a future release.
To remove this warning, do the following: 1) Pass option use_label_encoder=False w
hen constructing XGBClassifier object; and 2) Encode your labels (y) as integers s
tarting with 0, i.e. 0, 1, 2, ..., [num_class - 1].
  warnings.warn(label_encoder_deprecation_msg, UserWarning)
C:\Anaconda3\lib\site-packages\xgboost\sklearn.py:888: UserWarning: The use of lab
el encoder in XGBClassifier is deprecated and will be removed in a future release.
To remove this warning, do the following: 1) Pass option use_label_encoder=False w
hen constructing XGBClassifier object; and 2) Encode your labels (y) as integers s
tarting with 0, i.e. 0, 1, 2, ..., [num_class - 1].
  warnings.warn(label_encoder_deprecation_msg, UserWarning)
C:\Anaconda3\lib\site-packages\xgboost\sklearn.py:888: UserWarning: The use of lab
el encoder in XGBClassifier is deprecated and will be removed in a future release.
To remove this warning, do the following: 1) Pass option use_label_encoder=False w
hen constructing XGBClassifier object; and 2) Encode your labels (y) as integers s
tarting with 0, i.e. 0, 1, 2, ..., [num_class - 1].
  warnings.warn(label_encoder_deprecation_msg, UserWarning)
```

```
[22:45:38] WARNING: C:/Users/Administrator/workspace/xgboost-win64_release_1.3.0/s
rc/learner.cc:1061: Starting in XGBoost 1.3.0, the default evaluation metric used
with the objective 'binary:logistic' was changed from 'error' to 'logloss'. Explic
itly set eval_metric if you'd like to restore the old behavior.
[22:45:38] WARNING: C:/Users/Administrator/workspace/xgboost-win64_release_1.3.0/s
rc/learner.cc:1061: Starting in XGBoost 1.3.0, the default evaluation metric used
with the objective 'binary:logistic' was changed from 'error' to 'logloss'. Explic
itly set eval_metric if you'd like to restore the old behavior.
[22:45:38] WARNING: C:/Users/Administrator/workspace/xgboost-win64_release_1.3.0/s
rc/learner.cc:1061: Starting in XGBoost 1.3.0, the default evaluation metric used
with the objective 'binary:logistic' was changed from 'error' to 'logloss'. Explic
itly set eval_metric if you'd like to restore the old behavior.
[22:45:38] WARNING: C:/Users/Administrator/workspace/xgboost-win64_release_1.3.0/s
rc/learner.cc:1061: Starting in XGBoost 1.3.0, the default evaluation metric used
with the objective 'binary:logistic' was changed from 'error' to 'logloss'. Explic
itly set eval_metric if you'd like to restore the old behavior.
[22:45:38] WARNING: C:/Users/Administrator/workspace/xgboost-win64_release_1.3.0/s
rc/learner.cc:1061: Starting in XGBoost 1.3.0, the default evaluation metric used
with the objective 'binary:logistic' was changed from 'error' to 'logloss'. Explic
itly set eval_metric if you'd like to restore the old behavior.
[XGBClassifier - Test] accuracy_score: 0.806, preci_score: 0.583, recall_score: 0.
125, f1_score: 0.206, auc: 0.784,
 mse: 0.194, zero_one_loss_fraction: 0.194, zero_one_loss_num: 54.000, cv_score:
0.643
confusion_matrix:
 [[217   5]
 [ 49   7]]


[LightGBM] [Warning] feature_fraction is set=0.5, colsample_bytree=1.0 will be ign
ored. Current value: feature_fraction=0.5
[LightGBM] [Warning] lambda_l1 is set=0.01, reg_alpha=0.0 will be ignored. Current
value: lambda_l1=0.01
[LightGBM] [Warning] bagging_fraction is set=0.8, subsample=1.0 will be ignored. C
urrent value: bagging_fraction=0.8
[LightGBM] [Warning] lambda_l2 is set=10, reg_lambda=0.0 will be ignored. Current
value: lambda_l2=10
[LightGBM] [Warning] feature_fraction is set=0.5, colsample_bytree=1.0 will be ign
ored. Current value: feature_fraction=0.5
[LightGBM] [Warning] lambda_l1 is set=0.01, reg_alpha=0.0 will be ignored. Current
value: lambda_l1=0.01
[LightGBM] [Warning] bagging_fraction is set=0.8, subsample=1.0 will be ignored. C
urrent value: bagging_fraction=0.8
[LightGBM] [Warning] lambda_l2 is set=10, reg_lambda=0.0 will be ignored. Current
value: lambda_l2=10
[LightGBM] [Warning] feature_fraction is set=0.5, colsample_bytree=1.0 will be ign
ored. Current value: feature_fraction=0.5
[LightGBM] [Warning] lambda_l1 is set=0.01, reg_alpha=0.0 will be ignored. Current
value: lambda_l1=0.01
[LightGBM] [Warning] bagging_fraction is set=0.8, subsample=1.0 will be ignored. C
urrent value: bagging_fraction=0.8
[LightGBM] [Warning] lambda_l2 is set=10, reg_lambda=0.0 will be ignored. Current
value: lambda_l2=10
[LightGBM] [Warning] feature_fraction is set=0.5, colsample_bytree=1.0 will be ign
ored. Current value: feature_fraction=0.5
[LightGBM] [Warning] lambda_l1 is set=0.01, reg_alpha=0.0 will be ignored. Current
value: lambda_l1=0.01
[LightGBM] [Warning] bagging_fraction is set=0.8, subsample=1.0 will be ignored. C
urrent value: bagging_fraction=0.8
[LightGBM] [Warning] lambda_l2 is set=10, reg_lambda=0.0 will be ignored. Current
value: lambda_l2=10
```

```
[LightGBM] [Warning] feature_fraction is set=0.5, colsample_bytree=1.0 will be ign
ored. Current value: feature_fraction=0.5
[LightGBM] [Warning] lambda_l1 is set=0.01, reg_alpha=0.0 will be ignored. Current
value: lambda_l1=0.01
[LightGBM] [Warning] bagging_fraction is set=0.8, subsample=1.0 will be ignored. C
urrent value: bagging_fraction=0.8
[LightGBM] [Warning] lambda_l2 is set=10, reg_lambda=0.0 will be ignored. Current
value: lambda_l2=10
[LGBMClassifier - Test] accuracy_score: 0.806, preci_score: 0.750, recall_score:
0.054, f1_score: 0.100, auc: 0.814,
 mse: 0.194, zero_one_loss_fraction: 0.194, zero_one_loss_num: 54.000, cv_score:
0.580
confusion_matrix:
 [[221   1]
 [ 53   3]]
```

In [19]:

```
# 测试集：画roc/auc图
# （注：如果是要表达“选择最佳模型”，则在上面验证集评分后，选auc评分最高的【一个模型】，在这里单独画一条线出来即可，删去其他模型的测试集代码）
plt_roc_auc([
    (lr_test_result, 'lr', ),
    (tr_test_result, 'tr', ),
    (Gbdt_test_result, 'Gbdt', ),
    (Xgbc_test_result, 'Xgbc', ),
    (gbm_test_result, 'gbm', ),
], 'Test ROC')
```

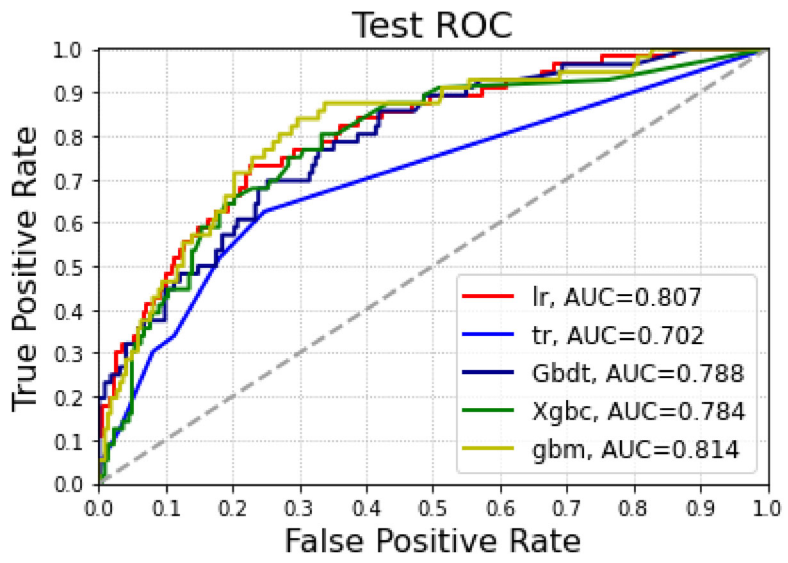

<Figure size 4800x9000 with 0 Axes>
